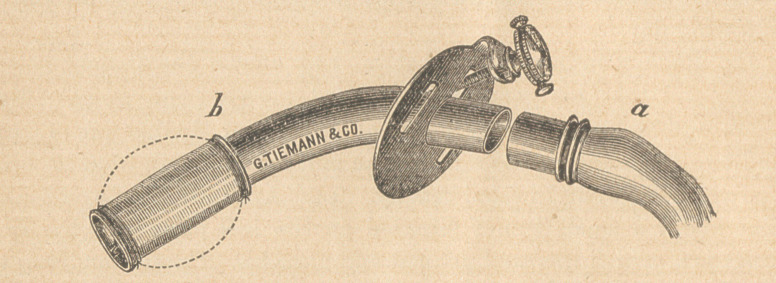# Extirpation of Cancer of the Face, Involving Inferior Maxillary Bone, Floor of the Mouth (Right Side), Submaxillary and Parotid Glands

**Published:** 1883-04

**Authors:** G. Wackerhagen


### EXTIRPATION OF CANCER OF THE FACE,

### INVOLVING INFERIOR MAXILLARY BONE, FLOOR OF THE MOUTH (RIGHT SIDE) SUBMAXILLARY AND PAROTID GLANDS.

Patrick S——, fifty-five years of age; farmer; born in Ireland, consulted me on the 6th of December last in reference to a tumor situated over the middle of the right lower jaw. He was suffering great pain from the growth; but otherwise seemed to be in good health, excepting that he had a very feeble pulse. He appeared to be anxious that some operation should be performed, as the tumor was rapidly increasing in size, and he found great difficulty in taking nourishment.

Upon examination I found the most prominent portion of the growth circular in shape, and about one and a half inch in diameter, with ulcerated surface. The remainder involved the inferior maxillary bone, right floor of the mouth, and the submaxillary and parotid glands.

After explaining to him the danger attending so formidable an operation, and the probability of its return, I told him that I would consult with his physician, Dr. Alex. J. Rooney, which I did on the following day.

Dr. Rooney informed me that he had known the patient several years, and that his general health had always been good. Three years ago he had attended him in an attack of double pneumonia; and two years ago he had removed an epithelial growth from his lower lip, when the wound healed readily. Last April Mr. Smith consulted him about a small tumor situated in the soft parts over the right lower jaw, which was well defined and movable. Upon examining the jaw, he discovered a decayed tooth, which he directed should be removed and the tumor painted with tincture of iodine. Under this treatment the tumor disappeared and did not return until about two months prior to his consulting me, when it commenced to grow rapidly, involving the surroundings in all directions.

On December 7th I visited the patient at his home in New Utrecht, and finding him still anxious for relief, determined to operate on the following day. The gentlemen present and assisting at the operation were Drs. Spier, N. Ford, Rooney and Silberman. Careful examination of the heart detected no organic trouble, though the pulse was remarkably feeble, but it improved during the administration of ether.

After placing a ligature upon the common carotid artery and internal jugular vein, I extended the incision upward around the lower portion of the tumor to the lower border of the inferior maxilla, following the lower border of this bone to its symphysis, and dividing the lower lip by a vertical incision. Commencing again at a point anterior to the tumor, the incision was carried above and around the upper and posterior portion, to the lower border of the jaw; thence around the angle and posterior border of the ramus, half way to the condyle. The incision was then continued around the posterior and lower border of the tumor, to the point where I first started. The tumor was then detached from the inferior maxillary bone, which was found to be extensively involved.

The hemorrhage being quite profuse, in spite of the previous ligation, it was considered advisable at this point to perform tracheotomy, and continue the anæsthetic through a tampon. The instrument devised by Dr. A. Gerster was used for this purpose, and it worked admirably and proved a great convenience. It would seem impossible to improve upon this instrument, and for the information of those who may not have seen it, the accompanying illustration is introduced.

The diseased portion of the lower jaw was now removed by sawing and cutting through near the symphysis and at the angle. Following this the floor of the mouth (right side), including the submaxillary glands, and finally the parotid gland, with all connecting diseased tissue, were thoroughly removed. After the application of carbolic acid solution to the wound, the skin was drawn over and united, with only slight tension.

The patient speedily rallied from the operation, and on the third day was able to sit up. There were no unpleasant symptoms from the ligation of the vessels. The temperature did not reach above 101°, but the pulse was very feeble and about 100. The nourishment consisted of beef-tea, brandy, milk and eggs. On the fourth day he refused the quinine and stimulants, and the attendants stated that it was with great difficulty he could be prevailed upon to take any nourishment. By the ninth day he refused all food, and I passed a tube into the stomach and injected some beef-tea and brandy. The patient resisted so much that it became necessary to have him held by the attendants. After this he took nourishment willingly, and in considerable quantity, until the twentieth day, when hemorrhage took place from the wound, the patient losing a pint of blood before it was discovered. Styptic cotton was applied by one of the attendants, and a compress placed over the wound. Upon removing the compress next morning, I could not discover the origin of the hemorrhage. After my departure the patient again refused nourishment.

About a week after the operation, the integument covering the wound commenced to slough, so that the wound was entirely uncovered. The tracheal incision also refused to heal. During the entire period there seemed to be no disposition toward granulation or healing ; and the patient becoming more and more exhausted, died on January 1st, twenty-five days after the operation. The dressings consisted of carbolized oil, iodine, iodoform, and nitric acid solution, but they seemed to have little effect in promoting the healing process. Friends would not permit pos-tmortem examination.—G. WACKERHAGEN, M.D., *in the Medical Record.*

## Figures and Tables

**Figure f1:**